# Untargeted and targeted metabolomics approaches to characterise, select and advance cassava pre‐breeding populations with enhanced whitefly tolerance

**DOI:** 10.1111/tpj.70233

**Published:** 2025-05-27

**Authors:** Laura Perez‐Fons, Adriana Bohorquez‐Chaux, Maria Isabel Gomez‐Jimenez, Luis Augusto Becerra Lopez‐Lavalle, Paul D. Fraser

**Affiliations:** ^1^ Department of Biological Sciences Royal Holloway University of London Egham UK; ^2^ Alliance Bioversity International and International Center for Tropical Agriculture (CIAT) Cali Colombia; ^3^ Present address: International Center for Agricultural Research in the Dry Areas (ICARDA) Rabat Morocco

**Keywords:** cassava, metabolomics, whitefly, breeding

## Abstract

Cassava (*Manihot esculenta* Crantz) provides food security for over 500 million people in Sub‐Saharan Africa (SSA). Whitefly (*Bemisia tabaci*) is a pest in this region that results in ca. 50% crop yield losses. Thus, it is important to develop approaches that will generate new varieties tolerant to this pest to advance food security in the region. Two parental cassava varieties, ECU72 tolerant to whiteflies and COL2246 a susceptible line, have been used to generate bi‐parental populations. The F1 generation has been screened for whitefly resistance, and progeny identified displaying enhanced tolerance. From designated F1 tolerant progeny, F2 families have been generated and phenotyped. The tolerance to whiteflies in the F2 population was further enhanced. Untargeted metabolomics was used to characterise whitefly susceptible and tolerant sub‐groups. PCA of the molecular features generated clustering of accessions into whitefly resistant and susceptible groups, and differentiating metabolite biomarkers were identified. The most significant metabolite marker for resistance is the chemical feature 316.0924. Although not consistent among all whitefly resistance sub‐groups, targeted LC–MS analysis revealed several pathways displaying perturbed levels. These include cyanogenic glycosides, apocarotenoids and the phenylpropanoid super‐pathway comprising hydroxycinnamic acids, flavonoids and proanthocyanidins. Thus, the generation of a bi‐parental population for whitefly tolerance/susceptibility enabled the identification of quantitative metabolite markers, the pathways contributing to tolerance, the underlying modes of action associated with resistance and the potential for the development of future high‐throughput low‐cost proxy markers. The approach also provides generic insights into future breeding strategies utilising bi‐parental progeny for the enhancement of traits.

## Significance Statement

A bi‐parental population has been created from cassava varieties ECU72 and COL2246, which are tolerant and susceptible to whitefly (*Bemisia tabaci*), respectively. Metabolomic characterisation of the F1 and F2 generations has revealed comparative metabotypes for whitefly tolerance, leading to metabolite biomarkers and sectors of metabolism contributing to underlying mechanisms of tolerance. These data provide a valuable resource for the incorporation of whitefly tolerance into cassava breeding programmes.

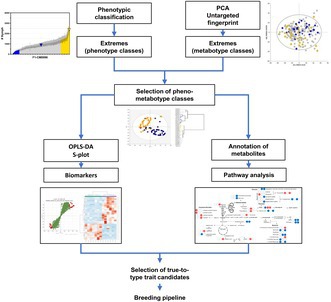

## INTRODUCTION

Cassava (*Manihot esculenta Crantz*) is a staple crop for many low and middle‐income countries populations. In Africa alone, over 500 million people are dependent on cassava and its products for food security. Its ability to withstand drought conditions and poor soils makes it a potential climate resilient crop (Parmar et al., 2017). Given these key socio‐economic criteria, significant investments have been made to advance cassava breeding as a means of addressing food/nutritional security. Genomic‐based approaches, including marker assisted selection (MAS) and genomic selection (GS) are the current approaches for accelerating cassava breeding programmes in Sub‐Saharan Africa (SSA) (Esuma et al., 2022; Okogbenin et al., 2007, 2012; Rabbi et al., 2022; Wolfe et al., 2017). These GS models and marker discovery studies have focused on exploiting SSA genetic resources and using phenotypic measurements mostly related to biomass and plant vigour (Wolfe et al., 2016, 2017, 2021). The diversity of the SSA cassava genetic pool used in breeding programmes is limited to multiple genetic crossings of existing germplasm (Ferguson et al., 2023; Rabbi et al., 2022; Reinhardt Howeler & Thomas, 2013; Xia et al., 2023), with the occasional introduction of diverse Latin American germplasm (Okogbenin et al., 2007, 2012).

Considering the level of investment in automation and computational power available to modernise cassava breeding programmes in SSA countries, comparatively little attention has been taken to address the foundational resources and tools that underpin traditional breeding programmes, namely genetic diversity, accurate phenotype determinations and robust trait discovery pipelines (Alemu et al., 2024). Although genetic tools directed to breeder implementation are prioritised, examples of discovery‐based outputs augmenting breeding strategies are now becoming evident. For example, the Cassava Source‐Sink (CASS) project provides a comprehensive understanding of the metabolic constraints and trade‐offs between dry matter and provitamin A traits in roots, as well as changes in the above‐ground vegetative biomass (Gutschker et al., 2024; Sonnewald et al., 2020). Tolerance to whitefly infestation has also been facilitated by the characterisation of Latin American diverse germplasm, whilst metabolomic and transcriptomic analysis has led to potential mechanisms of action being proposed (Irigoyen et al., 2020; Nye et al., 2023; Perez‐Fons et al., 2019). Infestation of cassava crops by whiteflies (*Bemisia tabaci*) can result in crop yield losses of up to 50% (Sam et al., 2024) and is thus a major target for cassava breeding. In the present work, phenotypic and metabolome characterisation of bi‐parental populations bred for whitefly resistance in cassava is presented. The aim of the study was to (i) use metabolite profiling as a complementary phenotypic tool for the selection of progeny, (ii) identify phenotypic markers at the metabolome level, (iii) identify underlying modes of action and (iv) indicate the use of these populations as pre‐breeding genetic resources.

## RESULTS

### Genetic resources and phenotypic classification

Two cassava accessions ECU72 and COL2246, with contrasting whitefly phenotypes, resistant and susceptible, respectively, have been characterised at metabolome and transcriptome level and a mechanism of action proposed (Irigoyen et al., 2020; Nye et al., 2023; Perez‐Fons et al., 2019). Both varieties were used to generate a number of bi‐parental crosses in order to: (i) assess phenotype heritability, (ii) obtain pre‐breeding material with a stable and transferrable whitefly resistance phenotype and (iii) traceable phenotype markers.

**Figure 1:**
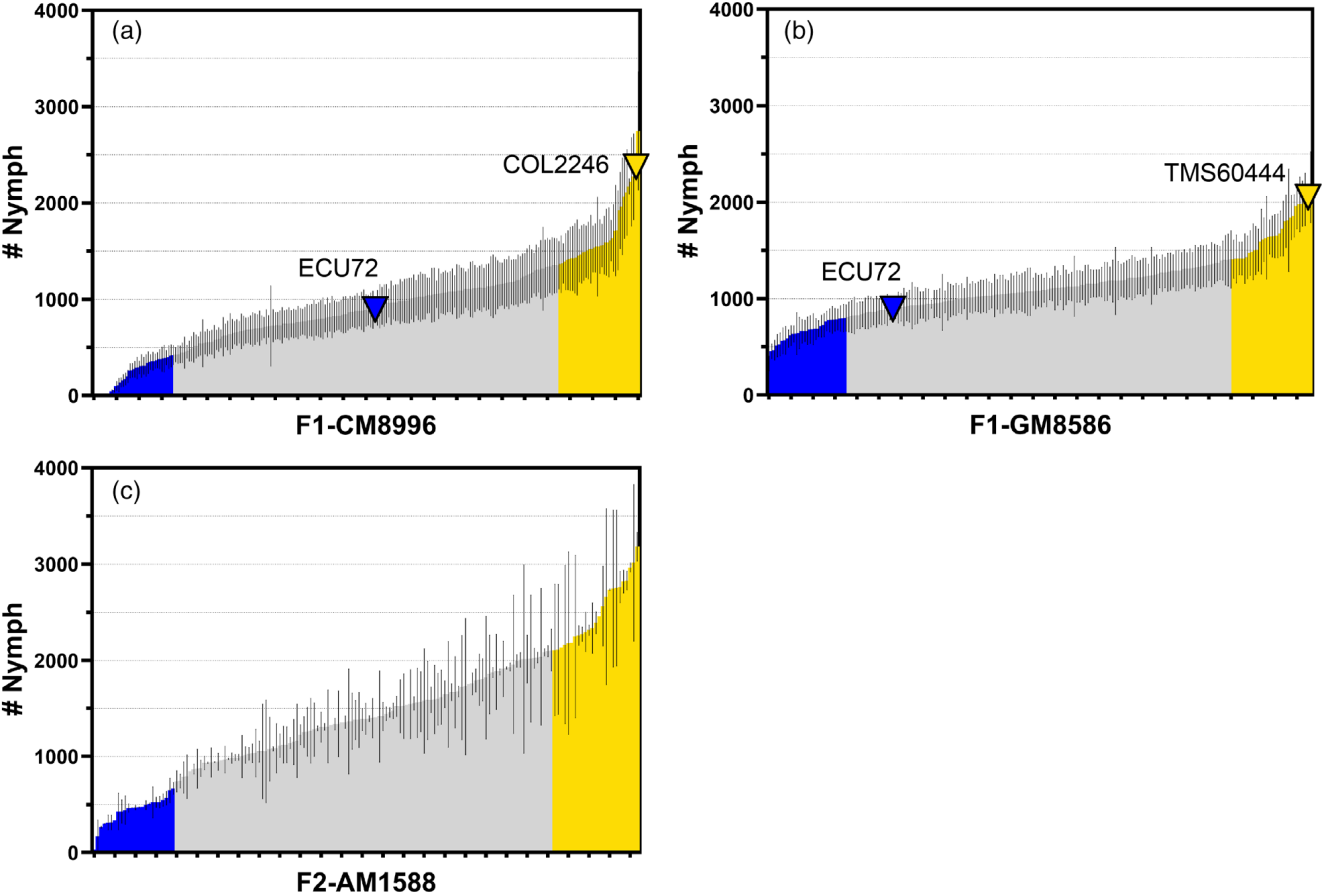
Phenotyping the F1 families CM8996, GM8586 and F2 AM1588 within the popultion using whitefly nymph counts. Percentiles 15 and 85 are coloured as extreme WF‐R (blue) and WF‐S (yellow) phenotypic groups, respectively. Parents are indicated with inverted triangle symbols and siblings as bars with standard error of mean of the different experiments. Siblings with intermediary phenotype are coloured in light grey.

A first generation (F1) of bi‐parental populations was established by crossing female flowers of the whitefly‐resistant (WF‐R) variety ECU72 and male flowers of susceptible (WF‐S) varieties COL2246 and TMS60444 from Latin American and African germplasm backgrounds, respectively. This strategy was defined by the characteristic male flower sterility of ECU72. The resulting F1 families, designated as CM8996 and GM8586 according to CIAT's internal nomenclature system, were then evaluated for whitefly phenotypes under multiple growing conditions using the automated nymph count system Nymphstar (Bohorquez‐Chaux et al., 2023). Both families presented a number of siblings showing enhanced WF‐R compared to that of the parent ECU72 (Figure 1a,b) when screened over multiple seasons, soil conditions and propagation methods. The average phenotypic determinations for each family compared to the parents show CM8996 being closer to the WF‐R parent and GM8586 being closer to the WF‐S parent (Figure S2). Segregating phenotypes were evident in both cases, with CM8996 presenting extremer whitefly phenotypes. Percentiles 15 and 85 of nymph counts were chosen as cut‐off points to designate extreme WF‐R and WF‐S phenotype classes, respectively. The difference between means of WF‐R and WF‐S classes in the CM8996 family was 1430 nymphs, whilst in GM8586, the difference was 1026 (Figure S1; Data S1). Additionally, the mean difference between WF‐R or WF‐S classes and the intermediary phenotype (WF‐I) was 662 and 768 nymphs in CM8996, but 440 and 586 in GM8586. In all cases, the differences between phenotype classes were highly significant (*P* < 0.0001).

Subsequently, advancement into the second generation of crosses (F2) was performed by crossing siblings with the highest WF‐R in CM8996 in order to produce pre‐breeding plant material with a stable/fixed WF‐R phenotype and reduced heterozygosity. Despite the male flower sterility of the female parent ECU72, sibling CM8996‐199 displayed enhanced flowering with viable male flowers, thus enabling a true‐F2 population by selfing CM8996‐199.

Phenotypic classification of true‐F2 AM1588 (Figure 1c) indicates that extreme WF‐R class differs significantly (*P* < 0.0001) from both WF‐I and WF‐S classes by 978 and 2068 nymph counts, respectively, and a mean's difference of 1090 nymphs separates significantly WF‐S from WF‐I classes (Figure S1; Data S2).

### Metabolome overview of bi‐parental crosses

An untargeted acquisition and data analysis approach was used for evaluating metabolome variation of bi‐parental populations F1 and F2 of ECU72. The number of chemical features extracted from the LC–MS analysis of each family was 3285 in CM8996, 4278 in GM8586, and 2284 in AM1588 (Data S3, S5, and S7). After filtering outliers and inconsistent missing values, the final untargeted data matrix used for subsequent analysis contained 2899 variables for CM8996, 4252 for GM8586 and 1384 variables for AM1588 (Data S4, S6, and S8).

**Figure 2:**
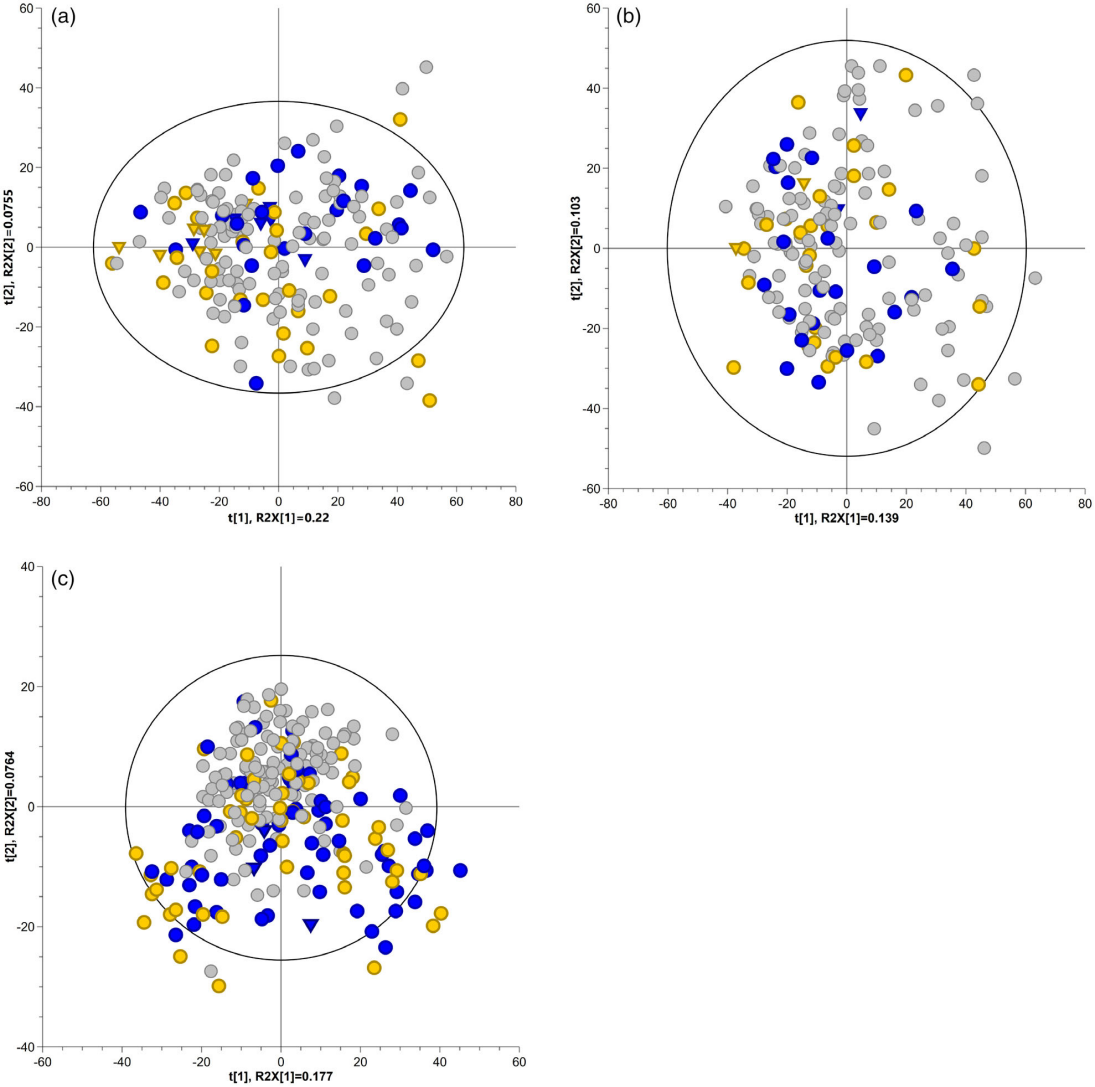
Principal component analysis of populations' metabolome. Score plot of untargeted LC–MS dataset of F1 family CM8996 (a), F1 family GM8586 (b) and F2 family AM1588 (c). Percentiles 15 and 85 coloured as extreme WF‐R (blue) and WF‐S (yellow). Parents represented with inverted triangle symbols and siblings as circles.

Components 1 and 2 of principal component analysis (PCA) explain 29.5% and 24.4% variation in the F1 families CM8996 and GM8586, respectively (Figure 2a,b), and 25.3% in the true‐F2 family AM1588 (Figure 2c). The score plots of both F1s present a wider spread of phenotypic classes WF‐R, WF‐S and WF‐I, whilst the F2 population AM1588 shows the extreme phenotypes (WF‐R and WF‐S) separating from the rest of the siblings, presenting an intermediary phenotype (Figure 2c). When extreme phenotype classes WF‐R and WF‐S were analysed independently by PCA and HCA, clusters within the WF‐R and WF‐S groups can be withdrawn. These groups were designated as extreme pheno‐metabotypes and used for comparative analysis and biomarker identification.

### Metabolite markers of whitefly resistance

**Figure 3:**
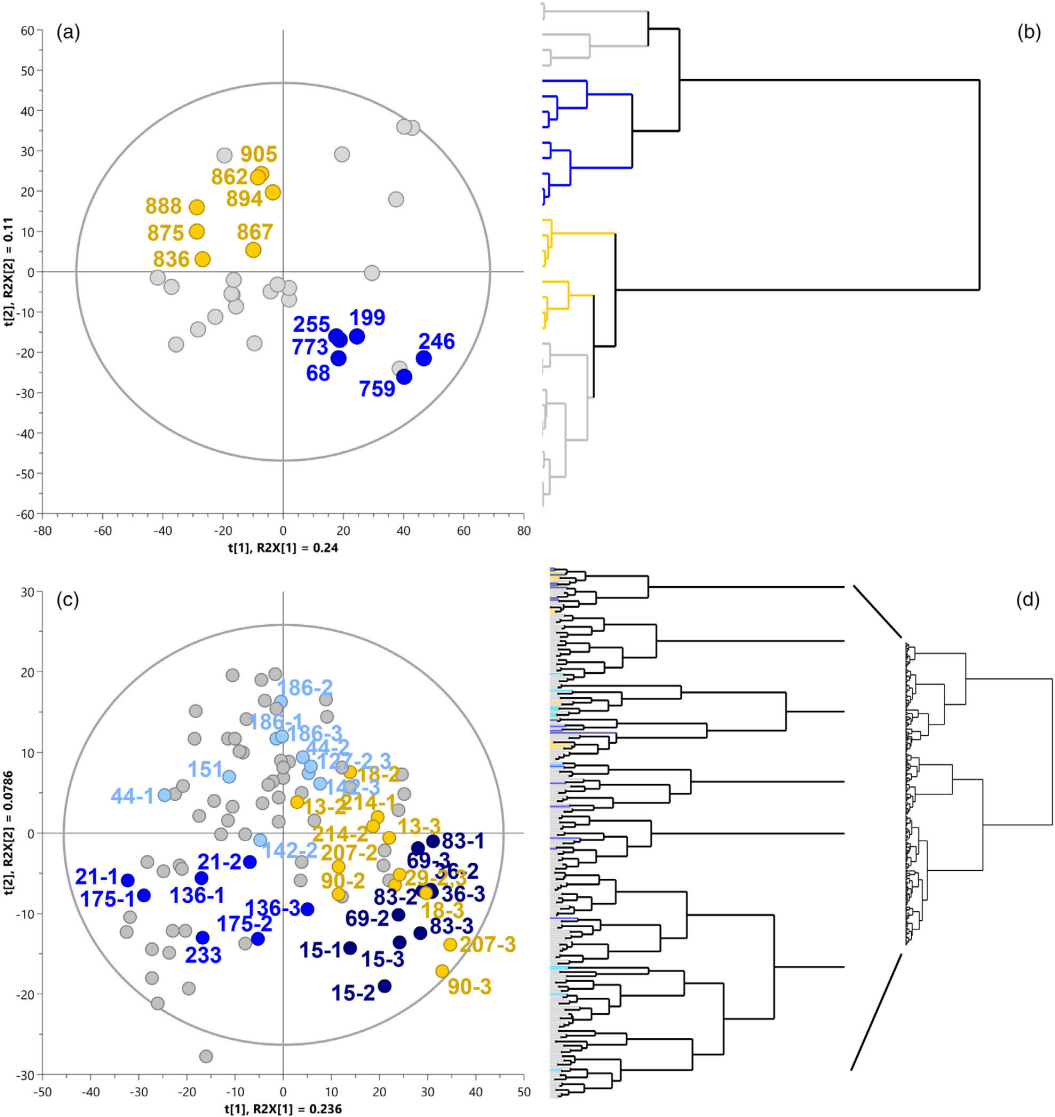
Multivariate analysis of the metabolomes from selected phenotypes (susceptible and tolerant) present in the F1 family CM8996 and F2 AM1588. Principal component analysis of selected extreme phenotypes of F1 family CM8996 (a) and F2 family AM1588 (c). The dendrograms of the populations' extreme phenotypic groups highlight the selected pheno‐metabotypes of F1 (b) and F2 (d) and coloured accordingly. The grey dots/bars represent those extreme phenotypes not selected as pheno‐metabotype groups; yellow grey/bars represent selected susceptible pheno‐metabotypes (WF‐S) and blue shaded dots/bars indicate selected resistant pheno‐metabotype groups (WF‐R). In the F2 family (c, d), dark blue represents whitefly‐resistant sub‐group 1 (WF‐R1), blue indicates whitefly‐resistant sub‐group 2 (WF‐R2) and light blue represents whitefly‐resistant sub‐group 3 (WF‐R3).

A range of supervised machine learning methods are available for biomarker identification in metabolomic studies (Anwardeen et al., 2023; Ghosh et al., 2020). In the present work, orthogonal partial least square discriminant analysis (OPLS‐DA) has been used to shortlist candidate metabolites associated with whitefly tolerance. The OPLS‐DA model descriptors obtained initially from the analysis of all the siblings selected based on the phenotype classification (15th and 85th quartiles of nymph count) of the F1 family CM8996 presented R2(cum) and Q2(cum) values of 0.484 and 0.0922, respectively, with only one predictive (P1) component and no orthogonal (O) components explaining the variation between and within both phenotypic classes. Similarly, the analysis of the extreme phenotypic classes of the F2 family AM1588 produce a model with R2(cum) fitting value of 0.978 and predictive Q2(cum) value of 0.444. However, the R2X(cum) of the predictive component P1 was substantially lower (0.012) compared to the sum of orthogonal components (0.444). Therefore, to refine/increase the accuracy of the model, the OPLS‐DA was performed using groups of extreme pheno‐metabotypes, that is, individuals of each phenotypic class clustering closely and opposite its phenotypic counterpart in the PCA score plot. In the F1‐CM8996, one metabotype sub‐cluster from each extreme WF‐R and WF‐S phenotypic class can be withdrawn (Figure 3a,b). The siblings in the WF‐R pheno‐metabotype class of CM8996 included 199, 246, 255, 68, 759 and 773, and those in the WF‐S pheno‐metabotype were 905, 894, 888, 875, 867, 862 and 836. In the F2‐AM1588, at least three sub‐groups of extreme WF‐R pheno‐metabotypes, WF‐R1, WF‐R2 and WF‐R3, were observed together with one group of WF‐S pheno‐metabotype (Figure 3c,d). The F2 family also included biological replicates of the selected extreme phenotypes, therefore grouping of replicates was an additional selection criterion applied in this case. Those siblings presenting high biological variation, that is, inconsistent clustering of biological replicates in the PCA score plot were not selected. The list of siblings in the AM1588 WF‐S sub‐group were 214, 207, 90, 13, 18 and 29. The metabotypes included in the different F2's WF‐R sub‐groups were 83, 15, 69 and 36 in the WF‐R1; 233, 175, 136 and 21 in the WF‐R2; 186, 127, 151, 142 and 44 in the WF‐R3.

**Figure 4:**
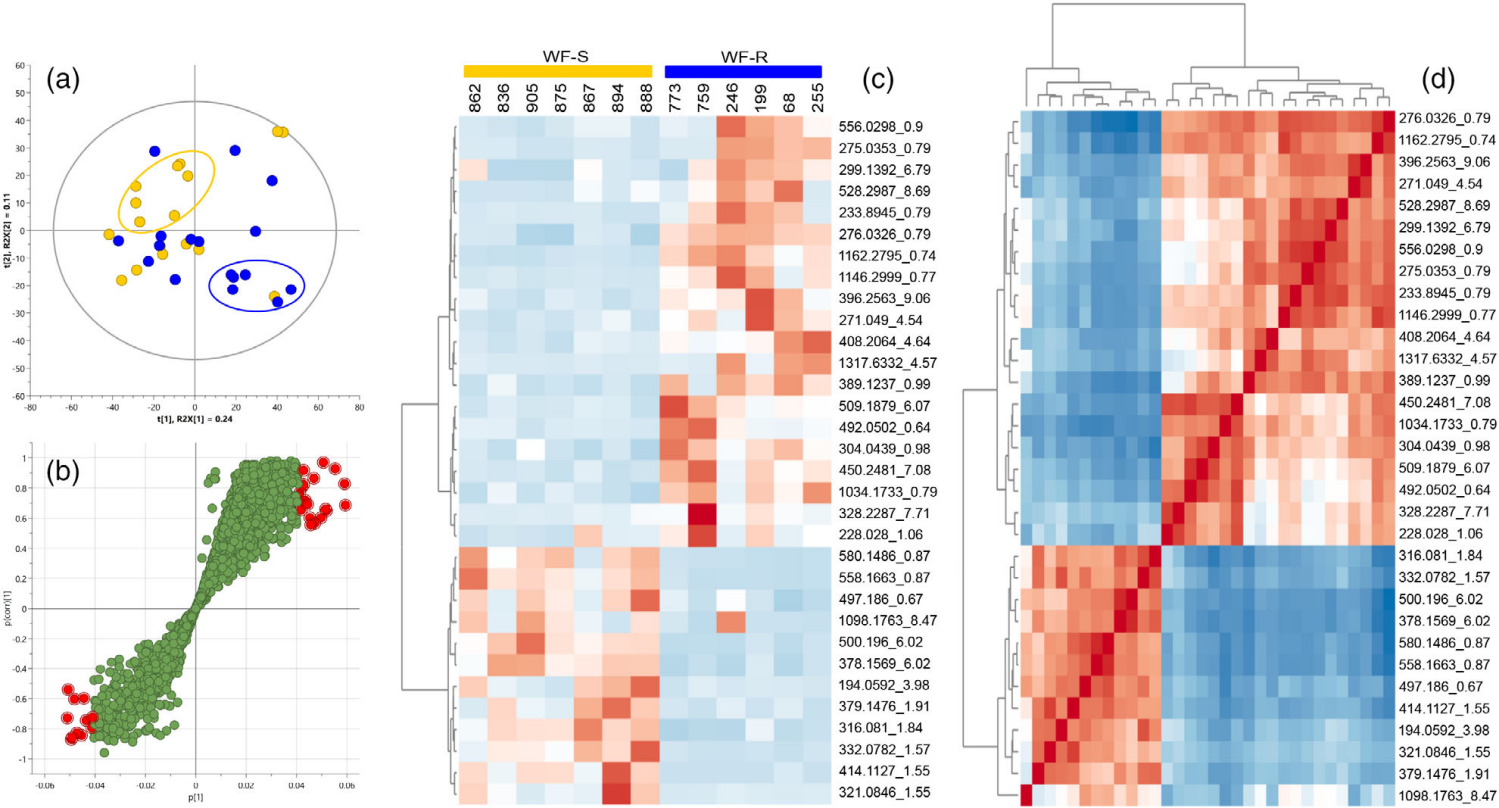
Example of data analysis workflow followed for the identification of metabolite markers differentiating pheno‐metabotype classes. (a) PCA scores plot of F1‐CM8996 extreme phenotypic classes, dark blue representing whitefly‐resistant (WF‐R) siblings and yellow dots representing whitefly‐susceptible (WF‐S) siblings. Clusters of extreme pheno‐metabotypes selected for OPLS‐DA analysis are circled. (b) Loadings S‐plot of the OPLS‐DA model using the selected WF‐R and WF‐S pheno‐metabotypes. Red dots indicate those chemical features presenting high correlation and covariance with phenotypic classes and selected as a list of markers. (c) Heatmap and dendrogram of chemical features retrieved from the S‐plot. (d) metabolite‐metabolite correlation (Pearson's) heatmap of selected markers. Detailed workflow of F2‐AM1588 pheno‐metabotypes sub‐groups is included in Figures S3–S5.

Following selection of extreme pheno‐metabotypes, an OPLS‐DA was applied to pairs of WF‐R and WF‐S pheno‐metabotypes in each bi‐parental family F1 and F2 to identify differentiating chemical features that correlate with whitefly resistance or susceptibility phenotype. The data analysis workflow is represented in Figure 4 using F1‐CM8996 extreme pheno‐metabotypes as an example, and the same rationale was applied to the pairs of pheno‐metabotypes found in the F2 (Figures S3–S5). In all cases, the predictive component of the model P1 defining class variation was higher than the orthogonal component O1 (or sum of them) defining the inherent class variation. Moreover, the cumulative R2 and Q2 values of the models representing, respectively, the level of phenotype variation explained or predicted by metabolome were over 0.8 (Data S9).

The loadings S‐plot of each OPLS‐DA model was performed to extract a list of candidate markers by selecting those loadings located at the extremes of the S‐tails (Figure 4b; Figures S3–S5b). In most cases, the correlation and covariance cut‐off values were 0.6 and 0.04, respectively. Some exceptions include a negative correlation cut‐off value of 0.5 in the CM8996 model, or a positive cut‐off variance of 0.06 in the WF‐R2 and WF‐R3 models of the F2 family AM1588. A list of 32 chemical features was obtained from the S‐plot of CM8996. Similarly, models comparing the different sub‐groups of WF‐R pheno‐metabotypes in the F2 family with the WF‐S class provided lists of 87, 58, or 64 candidates (Data S10). The heatmaps of the biochemical markers shortlisted showed reciprocal accumulation and correlation between WF‐R and WF‐S classes in each model (Figure 4c,d; Figures S3–S5c,d).

Only one chemical feature, 316.0924_5.34, was identified in all three WF‐R sub‐groups. The WF‐R2 and R3 shared the largest number of metabolite markers (13), whilst WF‐R1 shares two and four chemical features with WF‐R2 and R3, respectively (Figure S6).

### Targeted metabolite profile of whitefly resistant sub‐classes in F2 family AM1588

**Figure 5:**
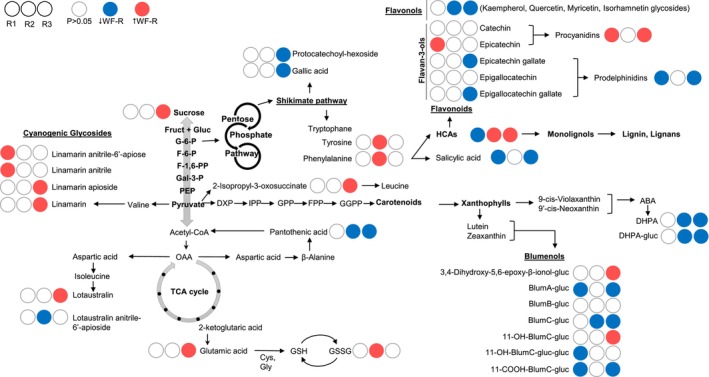
Pathway display illustrating the significant changes between F2's whitefly‐resistant sub‐groups WF‐R1, R2 and R3 compared to the whitefly‐susceptible (WF‐S) sub‐group. Red filled circles indicate concentration of metabolite is significantly (*P* < 0.05) higher in the WF‐R sub‐group, blue coloured circles indicate significantly (*P* < 0.05) lower concentration in the WF‐R sub‐group than the WF‐S group. List of acronyms and corresponding name of compounds is detailed in Data S14.

Using an in‐house metabolite library customised for cassava, the untargeted LC–MS data matrix was annotated. The metabolite LC–MS library included a wide range of chemical classes from primary metabolism (mono and disaccharides, amino acids, organic acids or fatty acids) to secondary metabolism (cyanogenic glycosides, hydroxycinnamic acids, hydroxybenzoates, flavonoids superfamily or apocarotenoids), among other miscellaneous compounds (Data S11). The resulting targeted data matrix contained 169 chemical features annotated (Data S12) and this was used as input for statistical analysis. Pair‐wise statistical comparison of WF‐R sub‐groups and WF‐S group of F2 family AM1588 was performed, and significant changes of metabolites mapped onto biosynthetic pathways as WF‐R/WF‐S ratios (Figure 5). In addition, detailed compositional differences are also summarised as heatmaps in Data S13.

The number of significant changes and the identity of the metabolites involved were different across the three WF‐R sub‐groups. The number of significant changes increased with the distance/position between the pheno‐metabotypes classes in the PCA plot in the following order: WF‐R1 < WF‐R2 < WF‐R3, with 14 significant metabolites differentiating WF‐R1 and WF‐S, 25 significant differences between WF‐R2 and WF‐S, and 61 significant differences between WF‐R3 and WF‐S.

The WF‐R1 presented higher levels of anitrile derivatives (non‐active) of cyanogenic glycosides, whilst active forms were significantly higher in WF‐R3, and no significant changes were detected in the WF‐R2 except for the anitrile form of lotaustralin apiose. Similarly, a different apocarotenoids profile was observed. All WF‐R sub‐groups display significantly lower concentrations of blumenols in general. The 11‐OH‐blumenol C glucoside and the 3,4‐dihydroxy‐5,6‐epoxy‐beta‐ionol glucoside were the only apocarotenoids significantly higher in one of the resistant sub‐groups (WF‐R3). Its oxidised by‐product 11‐COOH‐blumenol C glucoside, the precursor blumenol C glucoside and blumenol A glucoside were statistically lower in WF‐R3 and WF‐R1, but not in WF‐R2. In addition, both catabolites of abscisic acid (ABA), dihydrophaseic acid (DHPA) and its glucoside, were significantly lower in WF‐R2 and WF‐R3.

The phenylpropanoids super‐pathway comprising hydroxycinnamic acids (HCAs) and flavonoids was also significantly different across all WF‐R sub‐groups. Hydroxycinnamic acids involved in the monolignols biosynthesis were statistically higher in both WF‐R2 and R3 but lower in WF‐R1 when compared to the susceptible group. In contrast, flavonols and flavan‐3‐ols were generally lower in WF‐R3, and only epicatechin was higher in the WF‐R1 sub‐group. Polymerised forms of flavan‐3‐ols, procyanidins and prodelphinidins presented a contrasting trend, with significantly higher concentrations of procyanidins and lower levels of prodelphinidins in WF‐R1 and R3 but no statistical changes in WF‐R2. The WF‐R2 sub‐group presented significant reciprocal changes in HCAs and flavonols, as well as in the amino acids phenylalanine and tyrosine, which are the committed precursors of the phenylpropanoids biosynthesis.

## DISCUSSION

Previous studies demonstrate that different sectors of metabolism and a reprogramming of gene expression triggered by a contrasting phytohormone signalling are involved in the whitefly tolerance of cassava's natural variation (Irigoyen et al., 2020; Nye et al., 2023; Perez‐Fons et al., 2019). The wide range of molecular and biochemical events suggests an underlying complex genetic trait.

One of the strategies used by breeders to dissect complex genetic traits involving large and/or multiple chromosomic regions into minor QTLs that can be effectively transferred to farmers‐preferred or elite varieties in a stable manner is to create pre‐breeding material via recombinant inbred lines (RILs) or near isogenic lines (NILs) approaches. This enhances gene dosage of the trait, reduces epistasis and enables identification of reliable markers (Scott et al., 2020; Singh et al., 2015). These procedures are feasible in crops where botanical seeds are the propagation standards and genetic/biological resources and toolkits are well characterised (Acquaah, 2015; Singh et al., 2015). That is the case of maize (Liu et al., 2025; Teixeira & Guimarães, 2021), tomato (Perez‐Fons et al., 2014; The Tomato Genome Consortium, 2012), peppers (Liu et al., 2023; Lozada et al., 2022; Tripodi et al., 2021) or rice (Breseghello & Coelho, 2013), among many others. Cassava is a monoecious plant characterised by separate male and female flowers that mature asynchronously (protogyny) contributing to a high rate of male flower abortion and limited germination efficiency, challenges that are exacerbated by genetic and environmental factors influencing reproductive processes (Bandeira et al., 2021; Long et al., 2024; Njoku et al., 2015); thus, favouring clonal propagation as the standard method of choice. In addition, the frequency of natural cross‐pollination via insect vectors, usually bees, is higher than self‐pollination, which contributes to enhance the heterozygosity nature of the crop (Alves, 2002; Rodrmguez et al., 2023).

The present work successfully generated and characterised bi‐parental populations to develop clones exhibiting enhanced whitefly tolerance or resistance phenotypes. These clones also demonstrated reduced heterozygosity and refined genomic regions associated with the trait that could potentially serve as pre‐breeding material and true‐to‐type donors of the whitefly tolerance trait. The populations were characterised both phenotypically and at the metabolome level, with the two layers of information integrated and correlated gain deeper insights. The F1s families using ECU72 as trait donor confirmed heritability of whitefly tolerance across progeny, regardless of the genetic background – Latin American (COL2246) or African (TMS60444). Subsequent selfing crosses produced segregating families, with extreme phenotypes becoming more distinct. These observations suggest increased gene dosage effects or dominance driven by heterosis and epistatic interactions (Birchler et al., 2010; Fujimoto et al., 2018).

It has been reported that cassava's genetic diversity is correlated with metabolome composition (metabotypes) associated with specific trait/phenotypes (Perez‐Fons et al., 2023). Metabotypes – distinct profiles of metabolites within an organism – can provide insights into complex genetic interactions, such as epistasis, that traditional molecular markers might not fully elucidate. By analysing metabolic traits, researchers can uncover interactions between genes that influence phenotypic outcomes, offering a more comprehensive understanding of genetic architecture. For example, a study on *Arabidopsis thaliana* investigated the genetic basis of leaf molybdenum levels and identified significant statistical epistasis between a cis‐trans predictor pair affecting this trait. This interaction arose from high‐order linkage disequilibrium (LD) among four polymorphisms associated with molybdenum levels, suggesting that metabolic profiling can uncover complex genetic interactions that might be overlooked when relying solely on molecular markers (Zan et al., 2018). Similarly, a study on rice observed transgressive segregation for kilo‐grain weight in a recombinant inbred line population, finding that epistasis and complementary gene action adequately accounted for this phenomenon. This supports the idea that metabolic profiles can provide insights into genetic interactions that molecular markers alone might miss (Mao et al., 2011).

In this work, the extreme phenotypes of F1 and F2 populations also present distinctive metabolite profiles (metabotypes). When overlaying extreme phenotypes and metabotypes, only one cluster of whitefly resistance siblings can be identified in the F1 CM8996, whilst at least three sub‐groups of metabotypes are identified within the whitefly resistant cluster in the F2 AM1588. The statistical predictive model OPLS‐DA indicates that metabolome variation of the selected extreme pheno‐metabotypes explains accurately the phenotypic differences, with each phenotypic sub‐group presenting a specific metabolite profile composition. This could indicate that the genetic components (small‐QTLs) contributing to the complexity of the whitefly resistance trait start to segregate independently, thus providing valuable genetic resources for dissecting causal molecular and biochemical mechanisms of the trait, which would ultimately be translated into more accurate markers. The WF‐R1 sub‐group included the most resistant clones of the F2 family and yet presented the lowest significant differences when compared to the WF‐S group, whilst the WF‐R2 and R3 sub‐groups were markedly different from the WF‐S group. Despite the quantitative variability in pathways affected, there are common sectors of metabolism perturbed. For example, the phenylpropanoid super‐pathway. This supports previous findings (Perez‐Fons et al., 2019) that cell wall enhancement through altered precursor flux could be a component of the mechanism. Phytohormones, particularly abscisic acid levels, are altered in most of the resistant sub‐groups. This finding is in agreement with previous reports (Irigoyen et al., 2020; Nye et al., 2023). Finally, it is interesting that some blumenols are also affected as this opens new lines of investigation linked to the potential contribution/involvement by arbuscular mycorrhizal fungi (AMF) and stress tolerance (Wang et al., 2018).

**Figure 6:**
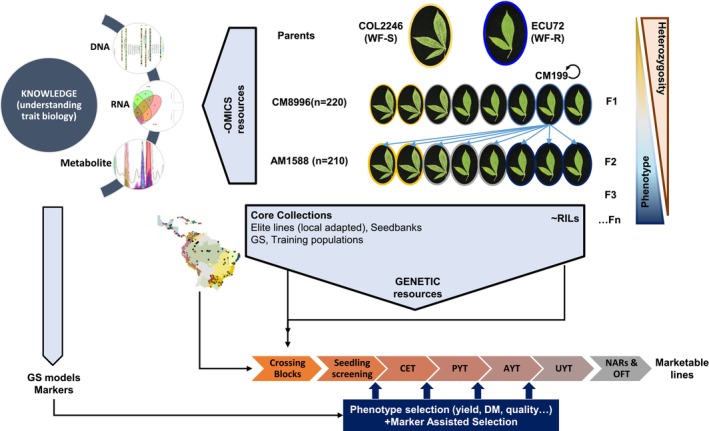
Schematic diagram of alternative breeding strategy integrating discovery/knowledge outputs into current cassava breeding pipelines. AYT, advanced yield trials; CET, clonal evaluation trials; DM, dry matter; GS, genomic selection; OFT, on‐farm trials; NARs, national agronomic research institutions; PYT, preliminary yield trials; RILs, recombinant inbred lines; UYT, uninform field trials.

Detailed analysis of the allelic variation among the genomic regions of interest will provide a selection of candidate genes that could refine the region further and contribute to the underlying molecular and biochemical mechanisms underlying the trait of interest. Incorporating the metabolomics and other‐omics at early stages of breeding programmes would also support the accuracy of genomic selection models by assisting the assembly and cross‐validation process of the training populations, typically used to build genomic prediction models and design breeding populations. The approach would ultimately reduce phenotyping costs and improve model accuracy (Colantonio et al., 2022). In the present study, whitefly tolerance has been used as the target trait, but the findings have generic implications that can benefit the current cassava breeding pipelines established in SSA through projects such as NEXTGEN (https://www.nextgencassava.org/) or RTB‐Breeding (https://rtbbreeding.cgiar.org/). For example, whereby the introgression of a donor F1 or F2 line with an enhanced phenotype into the recipient background (Figure 6). The trait will be stronger, presumably due to increased zygosity, but the potential to capture epistasis and heterosis is retained. In addition, the phenotypic strength will alleviate the inherited segregation from the heterozygosity of the recipient line. Synergistic to the approach are the potential for molecular and quantitative biochemical markers as well as systems‐level analysis for deciphering complex modes of action.

With the advent of AI and machine‐learning algorithms, metabolomics capacity to predict phenotype accurately is becoming closer to implementation in plant breeding (Fernie & Alseekh, 2022). Although the cost and high‐throughput metabolomics is still not comparable to sequencing technologies, the examples reported up to date suggest that models' accuracy built from metabolome data outperform those based on genomic information (Colantonio et al., 2022).

In summary, the present study has furthered valuable tools and resources for breeding new cassava accessions for whitefly tolerance. The approach used has contributed to elucidating underlying mechanisms associated with whitefly tolerance and has generic implications for added value to current cassava breeding programmes.

## METHODS

### Plant growing conditions

All bioassays were made at the CIAT experimental station. Shoot tips from *in vitro*‐grown cassava genotypes were excised and grown in 17 N rooting medium for 30 days in the cassava tissue culture laboratory at CIAT. These included whitefly susceptible genotypes (parentals COL2246 and TMS60444; check COL1468), one whitefly‐resistant genotype (parental ECU72), 220 progenies of the F_1_ CM8996, 198 progenies of the F_1_ GM8586 and 210 progenies of the F_2_ AM1588. The F_1_ families CM8996 and GM8586 were generated by crossing female flowers of whitefly‐resistant parent ECU72 and male flowers of whitefly susceptible accessions COL2246 and TMS60444, respectively. This is due to the characteristic male flower sterility of ECU72. The sibling 199 from the CM8996 presented viable male and female flowers, and it was therefore used for selfing and generating a true F2 family nominated as AM1588.

For hardening, the plants were sown in 2 L plastic bags with sterile substrate in a ratio of 20:10:1, black soil:sand:peat. Plants were grown in a glasshouse with an average temperature of 27°C under a photoperiod cycle of 12 h light:12 h dark.

### Mass rearing of *Aleurotrachelus socialis* and whitefly bioassays

The *A. socialis* colony was raised on *Manihot esculenta* var. COL1468 as previously described by Bohorquez‐Chaux et al. (2023). For the whitefly‐infestation bioassays, each 2‐month‐old cassava genotype was put into a mesh cage of 18 m large × 3 m wide × 3 m High to confine the whitefly adults after infestation in a glasshouse. Infestations were initiated by releasing ~22 000 adults of *A. socialis* into cages. When the adult whiteflies were removed at 3 dpi, the two youngest infested leaves, which are preferred by whiteflies for feeding and egg deposition, were tagged for future collection.

Three biological replicates were used for each genotype. Infested plants were placed in a randomised complete block design (RCBD) with each block containing a maximum of 100 plants (*N* = 100). Three plants per genotype (three biological replicates) were randomly allocated over the different blocks. To capture the effect of each life stage of the whitefly on the cassava plants, the sample collection time points were chosen to represent landmarks during the *A. socialis* life cycle. Leaf tissues from the 2‐month old cassava genotypes were harvested prior to infestation (time zero), 1 day post‐infestation (1 dpi; adult feeding and egg deposition) and 14 dpi (second and third instar feeding). After collection, leaves were frozen in liquid nitrogen and stored at −80°C. Subsequently, leaves collected were freeze‐dried (Christ LyoCube®) for 2 days and then shipped at ambient temperature in silica gel to analytical facilities at Royal Holloway University of London. The transit period did not exceed 3 days, and samples were stored at −80°C after arrival.

### Phenotyping

For the phenotyping of F1s and F2s and their respective parents and controls, a free‐choice experiment was carried out under greenhouse conditions, and an automated counting method called Nymphstar (Bohorquez‐Chaux et al., 2023) was used. With this approach, photographs of the infested leaves were analysed using the plugin, and data on nymph counts (NC) and area infested by nymphs were obtained.

In order to validate the consistency of the whitefly resistance phenotype, the infestation trials were performed under different growing conditions using plant material from *in vitro* culture or from stakes and/or micro‐stakes transferred from the field. Generally, *in vitro* grown material was planted onto poor soil (2:1 ratio of black soil:sand), and stakes were planted onto rich soil (3:1, black soil:sand ratio). The experiments performed over different seasons were as follows:

**Table:** 

Year	Cross	Propagation	Substrate
2013	CM8996	*In vitro*	Poor
2016	CM8996	Stake	Poor
2016	GM8586	Micro‐stake	Poor
2017	CM8996	*In vitro*	Rich
2017	CM8996	Stake	Rich
2017	GM8586	Stake	Rich
2018	CM8996	*In vitro*	Poor
2018	GM8586	*In vitro*	Poor
2020	AM1588	*In vitro*	Poor
2021	AM1588	Stake	Rich

### Metabolites extraction

Freeze‐dried tissue was ground to a fine powder using Qiagen TissueRuptor as described in Rosado‐Souza et al. (2019) and 10 mg was used for the extraction of metabolites as described in Perez‐Fons et al. (2019). Briefly, 700 μl of 50% v/v methanol was added, and the mixture was shaken for 1 h at room temperature. The addition of chloroform (700 μl) followed by centrifugation (3 min, 20 000g) allowed separation of semi‐polar and non‐polar compounds into the epiphase and organic phase, respectively. The semi‐polar extract was filtered with 0.45 μm nylon membranes, and the non‐polar extract was dried under vacuum. Both extracts were kept at −20°C until analysis.

### Untargeted metabolomics analysis by LC–MS

An aliquot of 95 μl of the semi‐polar extract (epiphase) was transferred to glass vials and spiked with 5 μl of internal standard (genistein at 0.2 mg ml^−1^ in methanol). Samples were kept at 8°C during analysis and volume injection was 1 μl.

For the analysis of the semi‐polar extracts, a C18 reverse phase column and a UHPLC‐ESI‐Q‐TOF system from Agilent Technologies (Santa Clara, CA. USA) were used. The analytical platform consisted of a 1290 Infinity II liquid chromatograph, a 6560 Ion mobility Q‐TOF mass spectrometer operating in Q‐TOF mode only and equipped with an Agilent Jet Stream (AJS) electrospray source. Data were acquired in MS mode from 100 to 1700 mDa under negative electrospray ionisation. Nebuliser and sheath gas temperatures were 325 and 275°C, respectively; flow rates of drying and sheath gas (nitrogen) were 5 and 12 L min^−1^, respectively, and nebuliser pressure was 35 psi. Capillary VCap, nozzle and fragmentor voltages were set up at 4000, 500 and 400 V. A reference mass solution was continuously infused to ensure mass accuracy calibration. Compounds were separated in a Zorbax RRHD Eclipse Plus C18 2.1 × 50 mm, 1.8 μm and 2.5% acetonitrile in water and acetonitrile as mobile phases A and B, respectively, both solvents containing formic acid (0.03% vol.) as an additive. The gradient started at 2% B for 1 min, increased to 30% B over 5 min, stayed isocratic for 1 min, followed by an increase to 90% B in 2 min. The washing step was maintained isocratic for 2 min, and initial conditions were restored for re‐equilibration for 3 min. Flow rate and column temperature were set at 0.3 ml min^−1^ and 30°C, respectively.

### Processing of untargeted metabolomics data files

Retention time alignment and extraction of chemical features of raw data files were processed with Agilent's Profinder (version 10.0) software using the Batch Recursive Molecular Feature Extraction mode. The following settings were selected to extract molecular features within a retention time (RT) range of 0.3–10 min: peak height threshold 1000 counts, RT tolerance ±0.2 min, mass tolerance 10 ppm, chlorine and formic acid adducts and water neutral losses were also considered. Only molecular features (MF) with matching scores higher than 70 and present in at least 15% of each sample group (QC and samples) were included in the final data matrix. This resulted in the detection of over 2000 MFE per population's dataset. Putative characterisation of chemical identity was inferred from accurate mass values calculated from mass‐to‐charge ratio (*m*/*z*) signals. Chemical formulae were generated using the following elemental constraints: C: 70; H: 140; O: 40; N: 10; S: 5; P: 3 (Ma et al., 2014) (https://pmn.plantcyc.org/CASSAVA/search‐query?type=COMPOUND&formula=C), formic acid and chlorine adducts and/or multiply charged species (*z* = 1, 2) were also considered. Those chemical formulas (up to 5) with the highest scores (based on mass difference and isotopic pattern fitting) were selected for blasting against ChemSpider and Dictionary of Natural Products chemical databases. Additionally, an in‐house library of cassava metabolites based on chromatographic parameters (retention time) and fragmentation pattern (MS/MS) (Perez‐Fons et al., 2019, 2023) was used to complement and validate findings of the putative identification pipeline described above.

### Statistical analysis and data analysis workflow

Batch correction of LC–MS untargeted data (extracted ion chromatogram peak areas) was applied using quality control samples (QC). In addition, normalisation against the area of the internal standard was performed to correct for instrument variation. Missing values were input by using the median value of each mass reported from the extraction chemical feature pipeline, and those presenting over 75% of missing values were excluded from analysis. The resulting data matrix was then used as input for multivariate analysis (PCA, HCA and OPLS‐DA) in SIMCA‐P v17 (Sartorius AG, Germany) and univariate analysis (*t*‐tests, anova and Pearson's correlation) in Prism v10 (GraphPad software LLC). Centring and pareto‐scaling were applied for multivariate analysis. Pairwise comparisons were performed by multiple two‐sample *t‐tests* assuming unpaired data, Gaussian distribution (parametric) and inconsistent standard deviation (Welch test). Multiple *t*‐test comparisons were corrected with the Holm–Sidak post hoc test setting a threshold value for significance (alpha) at 0.05. Heatmap displays were performed using MetaboAnalyst v5.0.

## AUTHOR CONTRIBUTIONS

LP‐F: Conceptualisation, performed metabolomics analysis and generated the corresponding dataset and related figures. LABL‐L and AB‐C designed bi‐parental populations, and AB‐C and MIG‐J performed the infestation trials, planting, phenotyping and collection of plant material. PDF and LABL‐L secured funding, devised the concept and provided resources. LP‐F, LABL‐L and PDF drafted the manuscript, and all authors participated in the discussion, interpretation of results and revision of the final version. PDF and LABL‐L acted as corresponding authors.

## CONFLICT OF INTEREST

The authors declare that they have no competing interests.

## Supporting information


**Figure S1.** Significant differences of phenotypic classes. anova unpaired Brown–Forsythe tests corrected for unequal variance (Welch's correction).
**Figure S2.** Heterosis effect. Bars represent mean and standard deviation of nymph counts. anova unpaired Brown–Forsythe tests corrected for unequal variance (Welch's correction).
**Figure S3.** Metabolite markers explaining phenotypic classification of F2's WF‐R1 sub‐group and WF‐S sub‐group.
**Figure S4.** Metabolite markers explaining phenotypic classification F2's WF‐R2 sub‐group and WF‐S sub‐group.
**Figure S5.** Metabolite markers explaining phenotypic classification F2's WF‐R3 sub‐group and WF‐S sub‐group.
**Figure S6.** Venn diagram of metabolite markers identified within the WF‐R class of the F2 family AM1588.

**Data S1.** Nymph count of F1 families CM8996 and GM8586.

**Data S2.** Nymph count of F2 family AM1588.

**Data S3.** Untargeted chemical features extracted from the LC–MS analysis of F1 CM8996. Raw data output obtained from Profinder v 10.0.

**Data S4.** Untargeted chemical features extracted from the LC–MS analysis of F1 CM8996. Processed data after QC correction and internal standard normalisation and used as input for multivariate and statistical analysis.

**Data S5.** Untargeted chemical features extracted from the LC–MS analysis of F1 GM8586. Raw data output obtained from Profinder v 10.0.

**Data S6.** Untargeted chemical features extracted from the LC–MS analysis of F1 GM8586. Processed data after QC correction and internal standard normalisation and used as input for multivariate and statistical analysis.

**Data S7.** Untargeted chemical features extracted from the LC–MS analysis of F2 AM1588. Raw data output obtained from Profinder v 10.0.

**Data S8.** Untargeted chemical features extracted from the LC–MS analysis of F2 AM1588. Processed data after QC correction and internal standard normalisation and used as input for multivariate and statistical analysis.

**Data S9.** Descriptors of OPLS‐DA models.

**Data S10.** List of markers obtained from the loadings S‐plot.

**Data S11.** Library of metabolite compounds used for annotation of chemical features.

**Data S12.** Targeted dataset of F2‐AM1588 including annotated compounds and relative quantification.

**Data S13.** Targeted dataset of F2‐AM1588 including *P*‐values (*t*‐test) and fold changes (ratios) using WF‐S group as reference.

**Data S14.** List of compounds' acronyms and corresponding names displayed in Figure 5.

## Data Availability

All relevant data supporting results presented are provided in supplemental files. Metabolomics data sets of raw (areas) and processed data (normalised), and phenotyping datasets are accessible in Mendeley Data: Perez‐Fons et al. (2025), ‘ACWP‐Cassava Mapping Populations Metabolomics’, Mendeley Data, V2, DOI: 10.17632/pdbjznbf2g.1.
