# Supplementary material for: Untargeted and targeted metabolomics approaches to characterise, select and advance cassava pre‐breeding populations with enhanced whitefly tolerance
Source: Plant J. 2025 May 27;122(4):e70233. doi: 10.1111/tpj.70233 (PMC12109377; doi:10.1111/tpj.70233)
Supplement: Supplementary file 1 — Figure S1. Significant differences of phenotypic classes. anova unpaired Brown–Forsythe tests corrected for unequal variance (Welch's correction). Figure S2. Heterosis effect. Bars represent mean and standard deviation of nymph counts. anova unpaired Brown–Forsythe tests corrected for unequal variance (Welch's correction). Figure S3. Metabolite markers explaining phenotypic classification of F2's WF‐R1 sub‐group and WF‐S sub‐group. Figure S4. Metabolite markers explaining phenotypic classification F2's WF‐R2 sub‐group and WF‐S sub‐group. Figure S5. Metabolite markers explaining phenotypic classification F2's WF‐R3 sub‐group and WF‐S sub‐group. Figure S6. Venn diagram of metabolite markers identified within the WF‐R class of the F2 family AM1588. [file TPJ-122-0-s015.pdf]

Metabolomics characterisation of cassava pre-breeding populations with enhanced whitefly tolerance.

Supplementary Figures

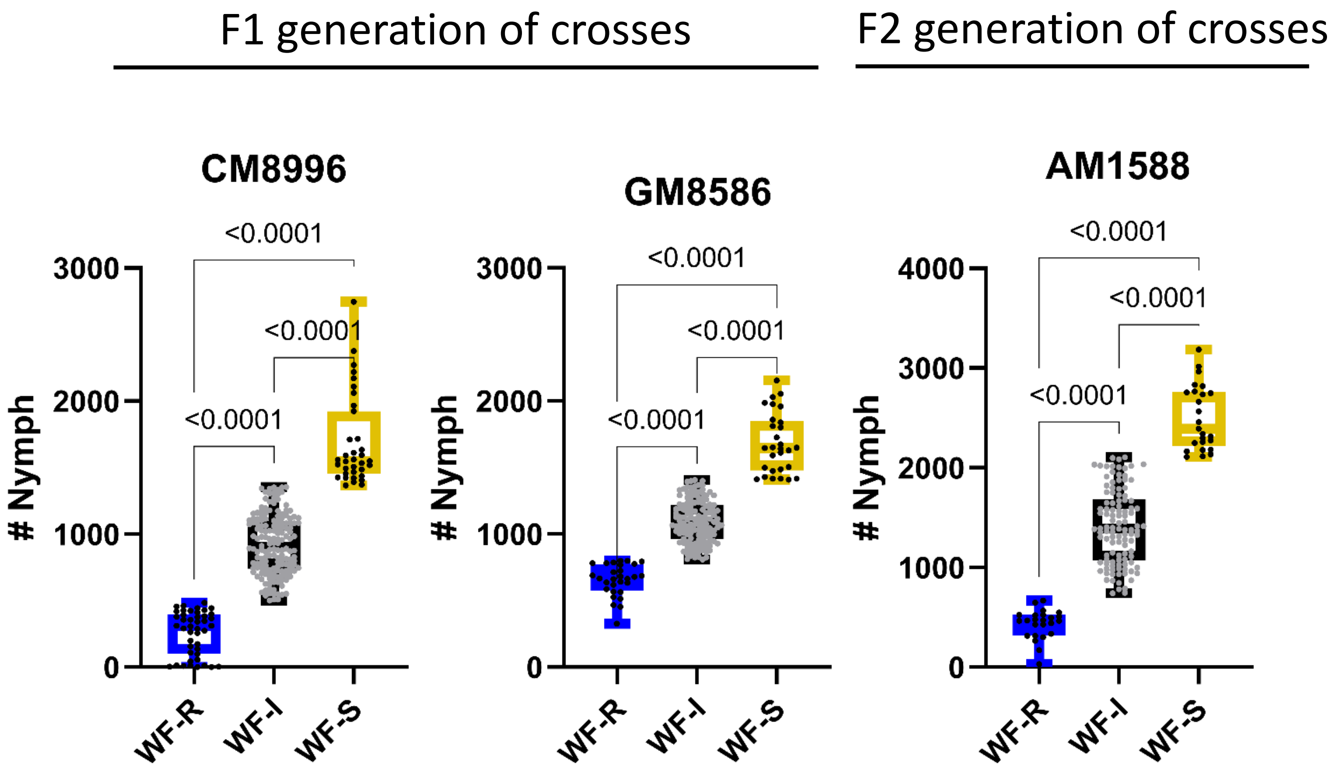

Supplementary Fig.S1: significant differences of phenotypic classes. ANOVA unpaired Brown-Forsythe tests corrected for unequal variance (Welch’s correction).

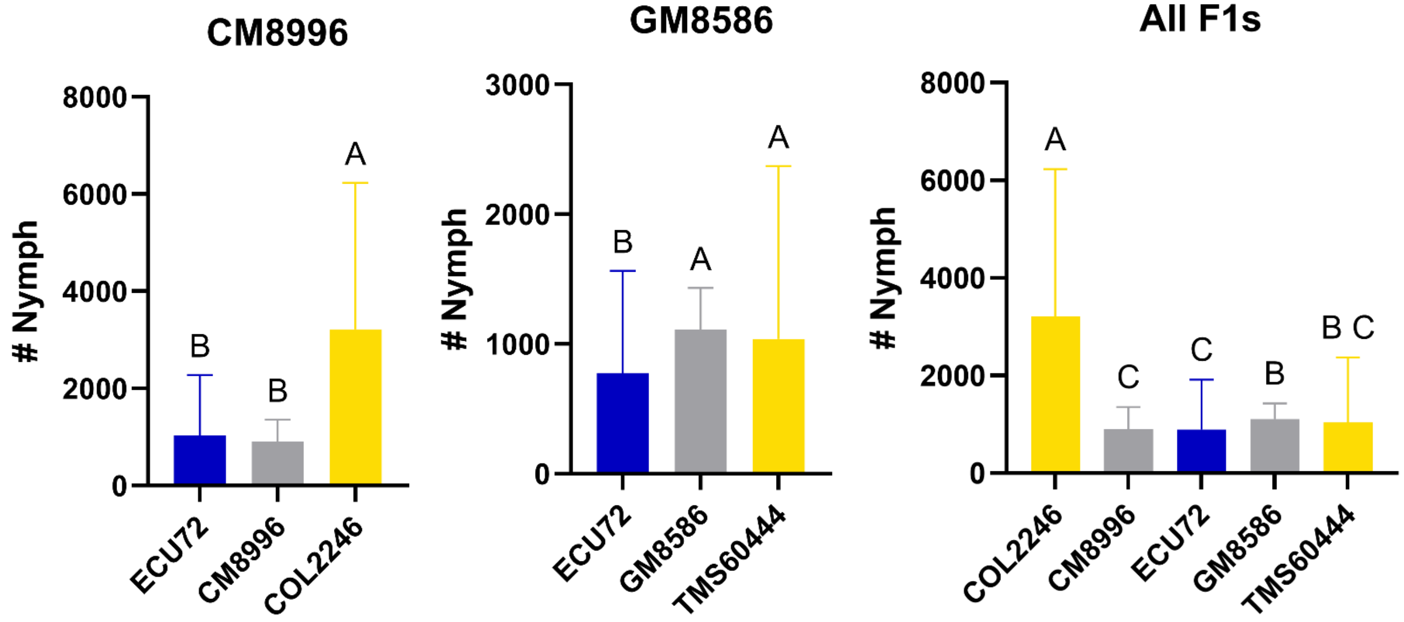

Table Analysed	**CM8996**	**GM8586**	**All F1s**
Brown-Forsythe ANOVA test			
F* (DFn, DFd)	103.7 (2.000, 297.1)	8.543 (2.000, 220.3)	94.52 (4.000, 356.9)
P value	<0.0001	0.0003	<0.0001
P value summary	****	***	****
Significant diff. among means (P < 0.05)?	Yes	Yes	Yes
Welch's ANOVA test			
W (DFn, DFd)	64.02 (2.000, 350.4)	21.88 (2.000, 287.5)	39.20 (4.000, 498.3)
P value	<0.0001	<0.0001	<0.0001
P value summary	****	****	****
Significant diff. among means (P < 0.05)?	Yes	Yes	Yes
Data summary			
Number of treatments (columns)	3	3	5
Number of values (total)	706	635	1341

Supplementary Fig.S2: Heterosis effect. Bars represent mean and standard deviation of nymph counts. ANOVA unpaired Brown-Forsythe tests corrected for unequal variance (Welch's correction).

Metabolite markers of extreme metabo/phenotypes

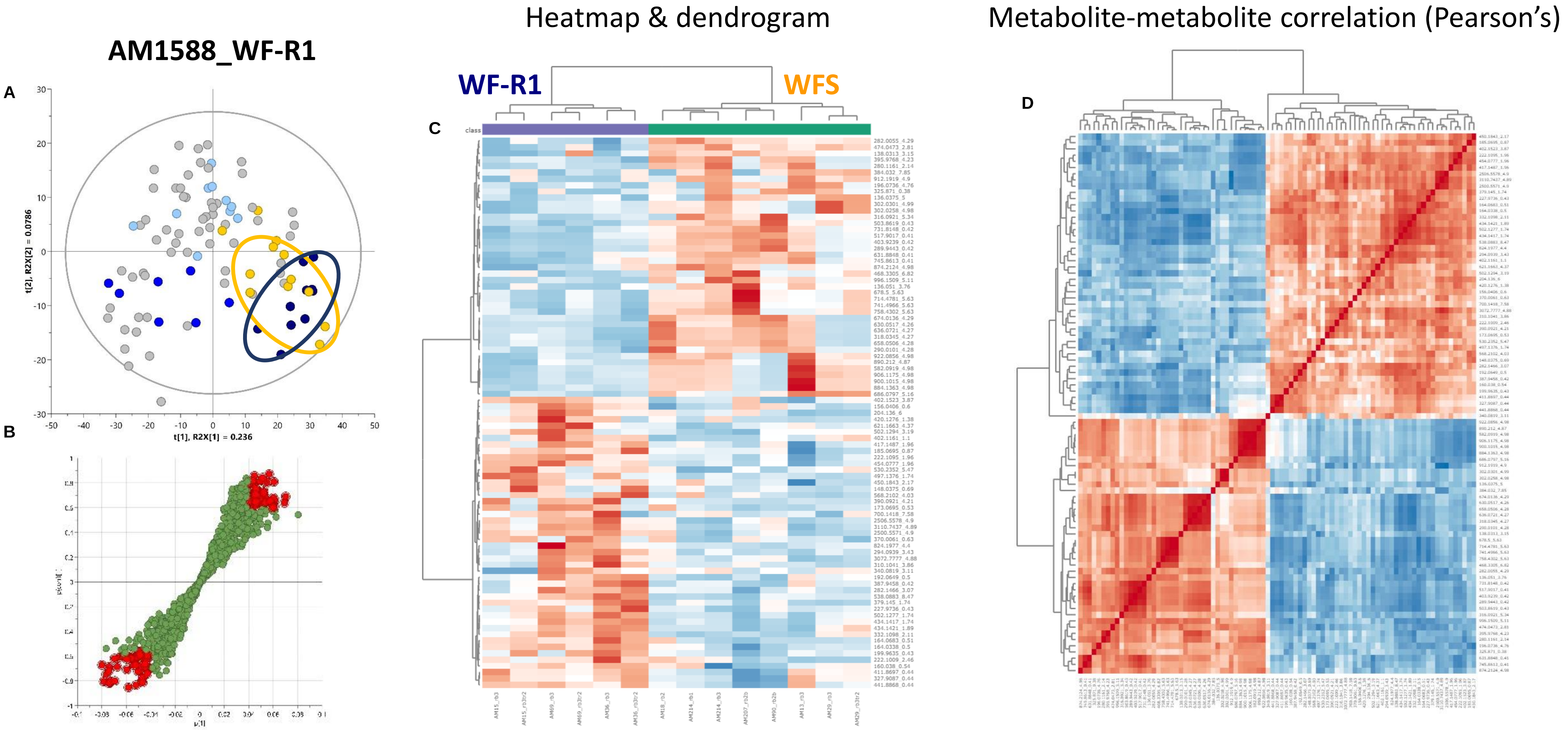

Supplementary Fig.S3: Metabolite markers explaining phenotypic classification of F2's WF-R1 subgroup and WF-S group.

Metabolite markers of extreme metabo/phenotypes

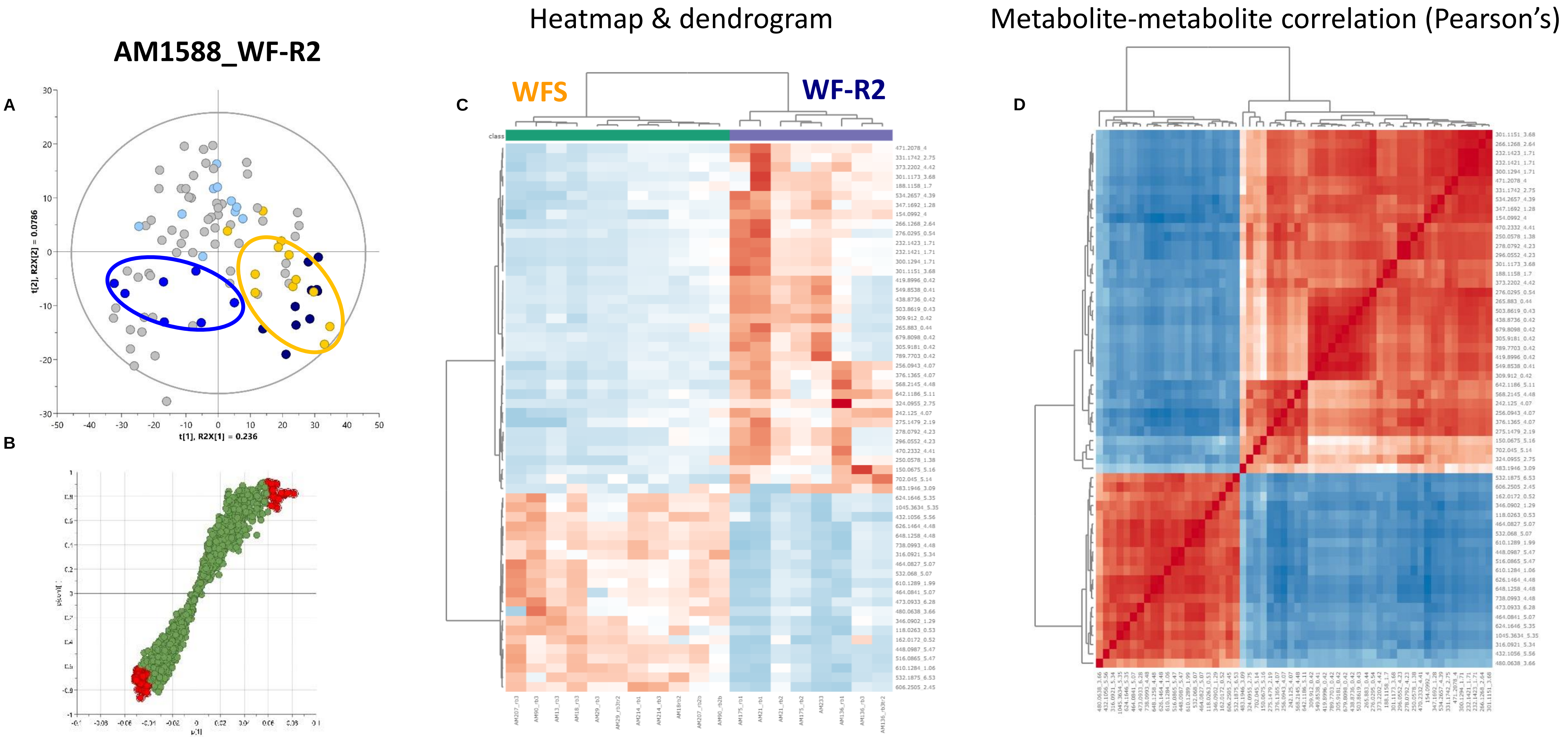

Supplementary Fig.S4: Metabolite markers explaining phenotypic classification of F2’s WF-R2 subgroup and WF-S group.

Metabolite markers of extreme metabo/phenotypes

AM1588_WF-R3

Heatmap & dendrogram

Metabolite-metabolite correlation (Pearson’s)

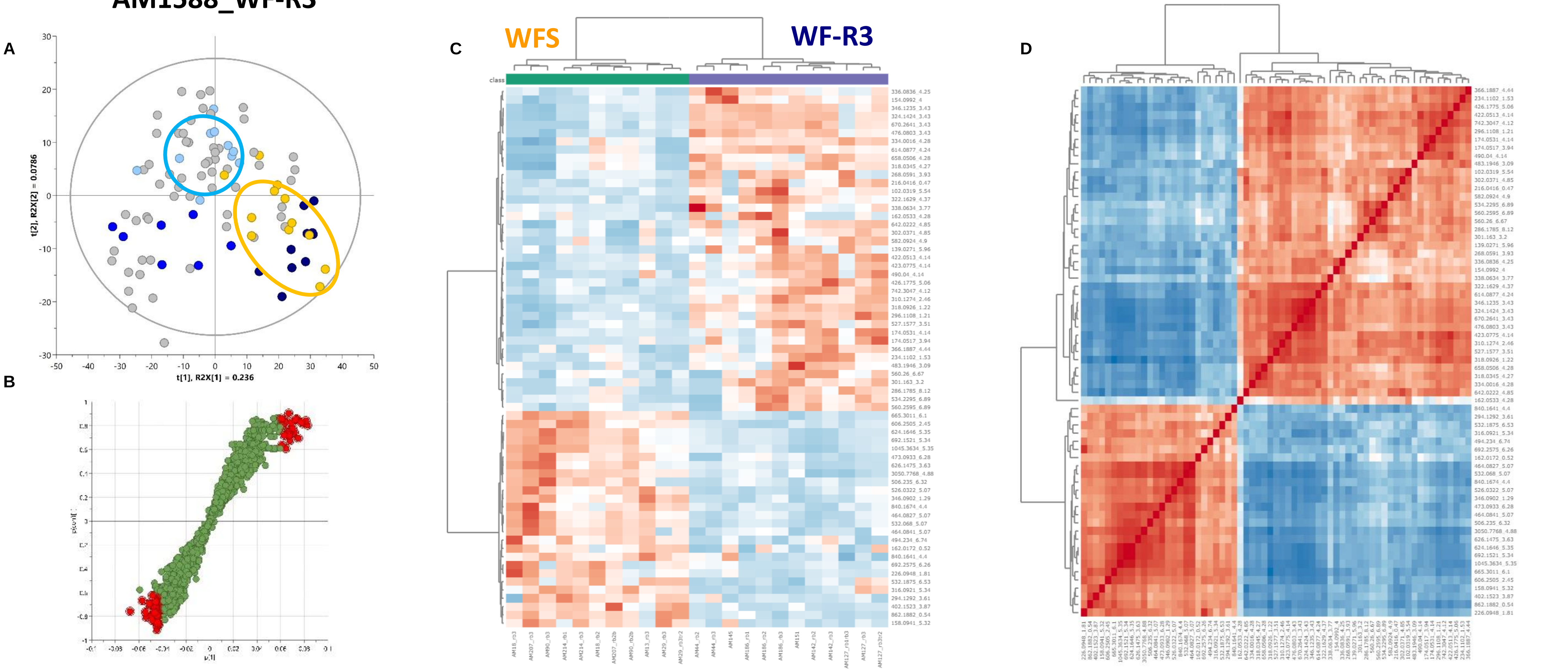

Supplementary Fig.S5: Metabolite markers explaining phenotypic classification of F2’s WF-R3 subgroup and WF-S group.

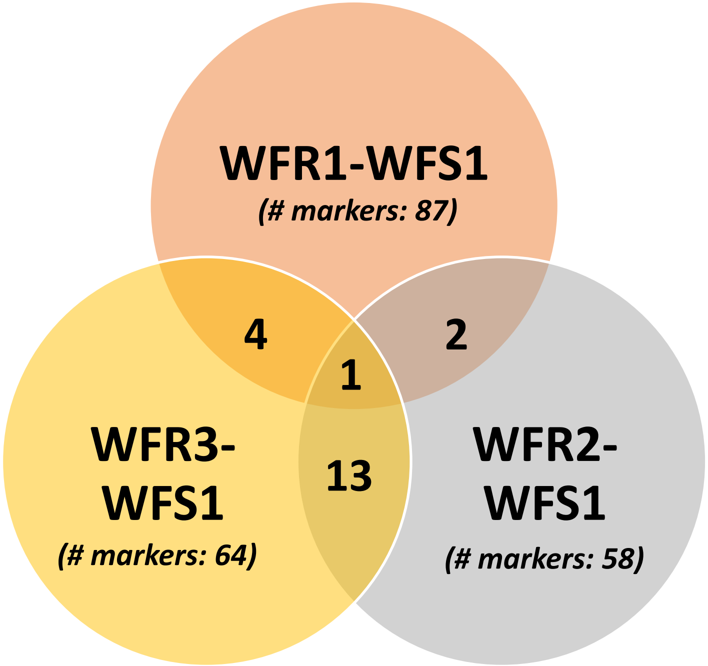

WFR1-WFR2	WFR1-WFR3	WFR2-WFR3
503.8619_0.43	402.1523_3.87	162.0172_0.52
316.0921_5.34	318.0345_4.27	346.0902_1.29
	658.0506_4.28	606.2505_2.45
	316.0921_5.34	483.1946_3.09
		154.0992_4
		532.068_5.07
		464.0827_5.07
		464.0841_5.07
		316.0921_5.34
		1045.3634_5.35
		624.1646_5.35
		473.0933_6.28
		532.1875_6.53

Supplementary Fig.S6: Venn diagram of metabolite markers identified within the WF-R class of the F2 family AM1588.
